# Olive Variety Classification and Prediction From 3D Morphology of Fruit and Stone: A Study Case on Five South Italy Autochthone Cultivars

**DOI:** 10.1002/fsn3.70797

**Published:** 2025-08-31

**Authors:** Laura Gargiulo, Antonio Gaetano Napolitano, Sabrina Maria Marsala, Anna Di Salle, Giacomo Mele

**Affiliations:** ^1^ Institute for Agricultural and Forest Systems in the Mediterranean (ISAFoM), Department of Biology, Agriculture and Food Sciences (DiSBA), National Research Council (CNR) Portici Italy; ^2^ Research Institute on Terrestrial Ecosystems (IRET), Department of Earth System Science and Environmental Technologies (DiTET), National Research Council (CNR) Napoli Italy

**Keywords:** authenticity, fruit anatomy, machine learning, plant phenotyping, traceability, variety distinctness, X‐ray microtomography

## Abstract

Accurate olive cultivar identification is critical for ensuring quality control and traceability in the olive oil industry. The International Olive Council (IOC) and the International Union for the Protection of New Varieties of Plants (UPOV) have established standardized protocols for varietal characterization. Over the past two decades, two‐dimensional image analysis techniques have been increasingly employed for olive variety identification, utilizing various morphological parameters and machine learning approaches. This study investigates olive varietal classification through three‐dimensional morphological analysis of fruits and stones using X‐ray microtomography. The research evaluates the discriminative power of different trait combinations using both Linear Discriminant Analysis (LDA) and Support Vector Machine (SVM) algorithms to contribute to an optimized protocol for cultivar identification. Five autochthonous olive cultivars from the Campania region (Southern Italy) were analyzed. A preliminary comparison of classification performance between continuous and discrete morphological olive data revealed superior effectiveness of the continuous ones. Integrating quantitative morphometric traits with selected visual discrete UPOV characteristics yielded optimal overall classification accuracy of 88.41% using LDA with 84.4% for Ravece, 81.5% for Ortice, 100% for Frantoio, 81.3% for Rotondella, and 90.9% for Minucciola olive varieties. The best variety prediction rates, based on an olive sample not used for training, were provided by SVM, obtaining 70.0% for Ravece, 87.5% for Ortice, 54.5% for Frantoio, 60.0% for Rotondella, and 66.7% for Minucciola. Quantification of varietal overlap through Bhattacharyya coefficients identified Ortice and Ravece as the most phenotypically similar varieties, while Rotondella and Minucciola exhibited the most distinctive fruit morphology. Notably, all varieties showed at least one misclassification with the Frantoio variety. Morphological analysis demonstrated that endocarp surface traits provided the most discriminative power, and internal cavity characteristics also contributed significantly to varietal differentiation. These findings suggest two key implications: potential updates of UPOV guidelines for distinctness evaluation protocols and promising applications in authenticity verification for high‐quality olive products.

## Graphical abstract

X‐ray micro‐CT allows accurate morphological olive fruit and stone phenotyping for quality monocultiivar olive oil traceability. Stone extremity traits mainly influence variety classification, but also internal ones. Bhattacharyya coefficient of trait discriminant functions helps in variety distinctness while Support Vector Machine provides best olive variety predictions also from few fruits.

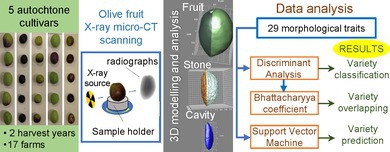

## 1 Introduction

The cultivated olive ( *Olea europaea* L. subsp. europaea var. europaea) is a globally significant oil crop, traditionally grown in the Mediterranean Basin, which remains the primary source of global production (International Olive Council; Mousavi et al. 2019). With over 2000 documented cultivars (Breton et al. 2009), the southern Italian region of Campania harbors a particularly rich genetic diversity, yielding olive oils of high typicality (Di Vaio et al. 2013).

Accurate cultivar identification is critical for the olive oil sector, ensuring traceability, geographical authenticity, and compliance with Protected Designation of Origin (PDO) standards (Likudis 2016; EEC No. 1143/2024). Many Italian PDO oils are monovarietal, derived from a single cultivar (e.g., Rotondi et al. 2013), making accurate cultivar discrimination essential for quality control and fraud prevention.

Genetic analysis for olive varietal identification offers high precision, but it requires specialized equipment and expertise (Martínez et al. 2018). Molecular markers (such as SSR, SNP, etc.) and new advanced biotechnological platforms have been used for genetic identity assessment and cultivar discrimination (Sebastiani and Busconi 2017; Gómez‐Rodrıguez et al. 2021; Montemurro et al. 2024), genetic approaches require expensive laboratory‐specific infrastructure and reagents, as well as specialized expertise in data interpretation and destructive sampling that prevents material re‐use (Pasqualone et al. 2016; Martínez et al. 2018; Blazakis et al. 2024). These limitations hinder their widespread adoption in resource‐limited settings such as small farms or regulatory field inspections.

Morphological characterization evaluates traits of the olive tree, flowers, leaves, fruits, and endocarps (stones), with endocarp morphology considered the most reliable for olive cultivar discrimination (Martínez et al. 2018; Manolikaki et al. 2022; Blazakis et al. 2024).

Structured protocols for olive cultivar characterization have been established by the International Olive Council (IOC) (Barranco et al. 2000) and the International Union for the Protection of New Varieties of Plants (UPOV). These protocols traditionally employ categorical descriptors of morphological traits of tree, shoot, leaf, fruit, and endocarp. However, recent UPOV Test Guidelines (TGP/8) now explicitly consider image analysis, suggesting revisions to conventional UPOV criteria (UPOV 2022). This reflects the growing recognition of quantitative morphometric approaches as a means to enhance objectivity and reproducibility in cultivar identification. On the other hand, with the increasing development of affordable high‐performance computer vision tools, morphological phenotyping can now provide a rapid, cost‐effective, and non‐destructive alternative or complementary method for cultivar identification and traceability (Mahanti et al. 2022; Liu et al. 2024).

Since 2000, 2D image analysis and machine learning techniques have been increasingly applied to olive varietal identification, often independently of IOC/UPOV standards. Early studies, such as Diaz et al. (2000), utilized machine vision for olive sorting based on colorimetric parameters, while Bari et al. (2003) compared a neural network approach and statistical classifiers to distinguish Mediterranean cultivars. Subsequent work by Vanloot et al. (2014) explored artificial vision and chemometrics for stone‐based varietal identification, overcoming traditional IOC descriptors. Advanced approaches, including morpho‐colorimetric parameters, elliptic Fourier descriptors, and surface texture analysis (Haralick's descriptors), were later integrated with linear discriminant analysis (LDA) to differentiate wild and cultivated olive populations (Piras et al. 2016). Further refinements incorporated IOC‐defined traits into image analysis, applying artificial neural networks (Beyaz et al. 2017), ANOVA and Tukey–Kramer tests (Blazakis et al. 2017), partial least squares discriminant analysis (PLS‐DA) (Martínez et al. 2018) and meta‐classifier approaches (Blazakis et al. 2024).

Despite these advances, 2D imaging may overlook critical 3D morphological characteristics essential for robust cultivar discrimination. Recognizing this limitation, Manolikaki et al. (2022) introduced 3D scanning for olive fruit phenotyping, while Ponce et al. (2019) employed image augmentation to infer 3D fruit sphericity. Moreover, Blazakis and Kalaitzis (2023) advocated direct 3D morphological analysis to minimize bias.

X‐ray micro‐computed tomography (micro‐CT) has emerged as a powerful tool for direct high‐resolution and comprehensive 3D phenotyping of small fruits and seeds (e.g., Lu et al. 2023, 2025; Gargiulo et al. 2019), offering high precision in quantifying internal and external 3D morphological traits. However, its application to olive cultivar identification remains unexplored, representing a significant methodological gap in olive fruit phenotyping research.

This study investigates the potential of 3D morphological traits derived from X‐ray micro‐CT imaging for olive cultivar discrimination. Fruits from five cultivars of the Campania region (southern Italy), sampled across two different harvest years from at least two distinct farms per cultivar, were analyzed. LDA was applied to evaluate the contribution of different morphological traits to variety classification, while a Support Vector Machine (SVM) learning approach evaluated the overall prediction performance.

## 2 Materials and Methods

### 2.1 Plant Material

Drupes (fruits) and endocarps (stones) from five cultivars (Ravece, Ortice, Frantoio, Rotondella, and Minucciola) of *Olea europaea* L. were investigated in this study. Sampling was conducted during October and November of 2020 and 2021 across multiple olive orchards in the Campania region of southern Italy (Table S1). These cultivars are recognized for their use in high‐quality mono‐cultivar oil production (Rotondi et al. 2013).

Fruits were manually harvested at phenological growth stages 85–89 (Sanz‐Cortés et al. 2002), corresponding to full fruit development and a maturity index ≥ 4 (e.g., Mailer and Gafner 2021). Post‐harvest, olives were stored in a climate‐controlled environment (7°C) and weighed using a precision balance prior to 3D imaging via X‐ray micro‐CT. A total of 121 fruits were scanned, with sample sizes per cultivar ranging from 11 to 35 (Supplementary Figure 1).

### 2.2 X‐Ray Micro‐CT

The X‐ray micro‐CT scans of individual olive drupes were performed using a desktop microtomograph (Bruker Skyscan 1272; http://bruker‐microct.com/products/1272.htm). It is equipped with a cone‐beam X‐ray source with adjustable energy (20–100 kV) and accommodates samples up to 6.5 cm in diameter and 7.2 cm in height. To optimize magnification and scanning duration, each drupe was positioned horizontally or obliquely during imaging.

For 3D imaging, the X‐ray source was set at 80 kV voltage and 124 μA current, with a 0.5 mm aluminum filter placed between the source and sample to filter the lowest energy range of X‐rays. The source‐to‐sample distance varied between 130 and 260 mm. Approximately 300 projection images were captured over a 180° rotation (0.7° increment per step), achieving a voxel size of 15–27 μm, depending on geometrical magnification and olive size. Total scan time per sample was ~15 min.

### 2.3 3D Image Processing and Analysis

The two‐dimensional X‐ray projection images acquired from each micro‐CT scan were reconstructed into three‐dimensional volumes using NRecon software (v2.2.0.6; Bruker MicroCT, Kontich, Belgium). Reconstruction was performed via a filtered back‐projection algorithm (Xiao et al. 2003), with ring artifact correction (50%) and beam hardening correction (100%) applied to optimize image quality. The reconstruction process required approximately 40 s per dataset.

**FIGURE 1:**
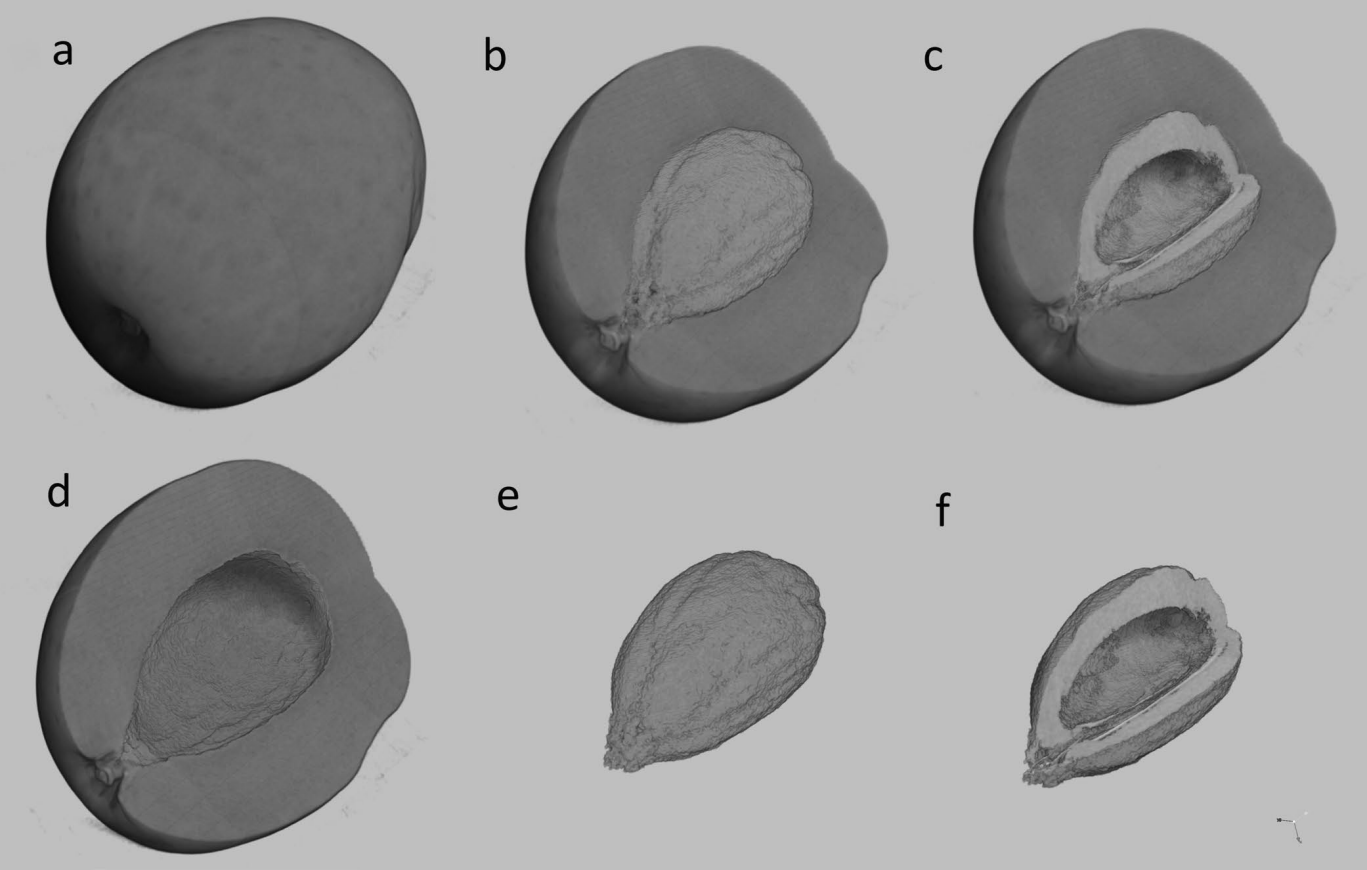
3D reconstruction of an olive and its internal parts with X‐ray micro‐CT. (a) Whole fruit; (b) sectioned mesocarp and stone; (c) sectioned mesocarp and sectioned stone with its internal cavity; (d) sectioned mesocarp without stone; (e) whole stone; (f) sectioned stone with its internal cavity.

DataViewer software (v1.7.0.1; Bruker) was employed to define volumes of interest (VOIs) and reorient reconstructed volumes along the vertical axis. Tissue segmentation was subsequently performed using Otsu's thresholding method (Otsu 1979) to differentiate mesocarp (fruit tissue) from endocarp (stone tissue) (Figure 1).

Image thresholding and processing were performed using CTAn software (v1.20.8; Bruker MicroCT, Kontich, Belgium).

Quantification of morphometric traits was conducted on the reconstructed images by applying the so called “Object‐based Image Analysis” (OBIA) approach (e.g., Blaschke 2010) on the different segmented fruit parts: whole fruit (Figure 1a), stone (Figure 1e), stone cavity (Figure 1f). Image ProPremier 3D software, version 9.2 (www.mediacy.com) was used for the image analysis.

Definitions of the morphometric traits of the whole fruit, stone, and stone cavity and of the UPOV characteristics of fruit and stone are reported in detail in Appendix A.

### 2.4 Data Analysis

All univariate and multivariate analyses were conducted on both morphometric traits and UPOV characteristics (notes). Differences among varieties were assessed using Tukey's HSD test (*p* < 0.05) as the univariate approach. Pearson correlation coefficients were computed, along with their corresponding *p*‐values. For multivariate analysis, linear discriminant analysis (LDA) was performed to discriminate among the five olive varieties, reducing the high‐dimensional variable space to four dimensions. This was applied using different predictor combinations (traits and/or notes). Specifically, LDA was conducted separately for each predictor type: those referred to whole fruits, those referred to stones, and those of internal stone cavities. Prior to multivariate analysis, potential outliers within each variety group were evaluated using Mahalanobis distance.

The Bhattacharyya distance (*D*_*B*_) (Bhattacharyya 1943) was calculated between all pairwise combinations of varieties in the DF^4^ domain:

(1) $$D_{B}\left(v_{1}v_{2}\right)=\frac{1}{8}{\left(\mu_{1}-\mu_{2}\right)}^{T}\Sigma^{-1}\left(\mu_{1}-\mu_{2}\right)+\frac{1}{2}\ln\left(\frac{\text{detΣ}}{\sqrt{\det\Sigma_{1}\det\Sigma_{2}}}\right)$$

where, (*v*_1_, *v*_2_) is a given couple of varieties, μ_1_ and μ_2_ are the four‐element vectors of the average values of the four discriminant functions of the olive varieties *v*_1_ and *v*_2_, ∑_1_ and ∑_2_ are the variance/covariance matrices of the DF^4^ distributions for the two olive varieties, while

$$\sum =\left(\sum_{1}+\sum_{2}\right)/2$$

Then the Bhattacharyya coefficient *BhC*,

(2) $$\mathrm{BhC}\left(v_{1},v_{2}\right)=\exp^{-1}\;D_{B}\left(v_{1},v_{2}\right)$$

was calculated as a measure of overlap between the two multivariate datasets of the DF^4^ distributions of *v*_1_ and *v*_2_.

Additionally, the potency index for each predictor was computed to rank predictor importance in the LDA (Malarvizhi and Geetha 2015). The potency index for each predictor was derived by summing the four potency values obtained by multiplying the squared loading of each predictor by the corresponding Eigen value of each discriminant function (see File S2).

To comprehensively evaluate the prediction potential of fruit and stone morphological data for olive variety classification, a SVM learning model with a linear kernel was implemented. The model was trained on 70% of the variety‐labeled dataset, with the remaining 30% untagged reserved for validation. For each variety, the representation of both harvest years and different farms was ensured, and the individual olives selected for training and validation are specified in File S1.

Tukey tests, Mahalanobis distance measurements, and LDA were carried out using SPSS software version 28.1.1 (www.ibm.com), while Pearson correlation coefficients with *p*‐values were determined using Sigmaplot software version 13.0 (www.systatsoftware.com). Bhattacharyya distance was calculated using Excel. The SVM model was applied using the “scikit‐learn” library of machine learning in Python (https://scikit‐learn.org/).

**FIGURE 2:**
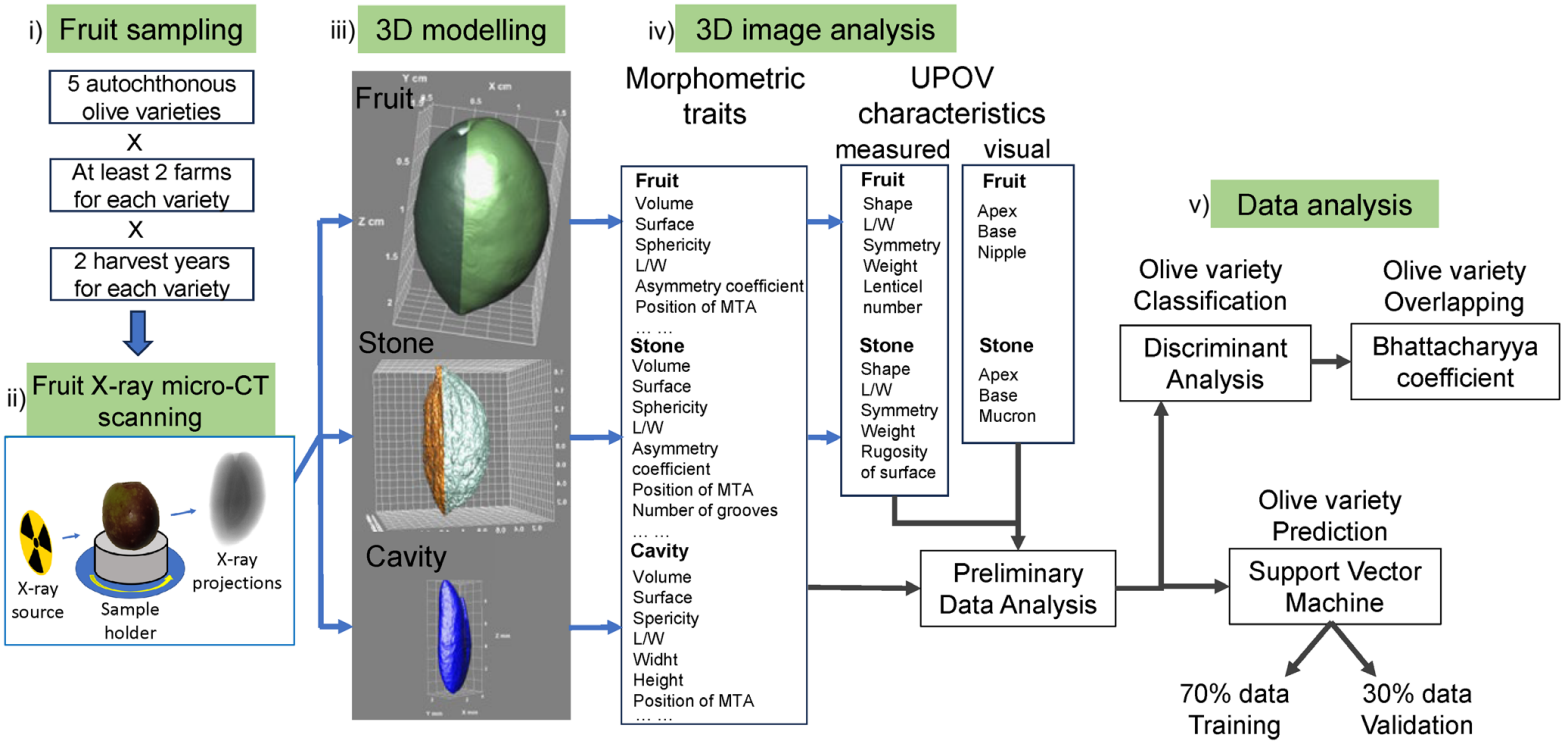
Flowchart of the experimental design. (i) Fruit sampling, (ii) fruit X‐ray micro‐CT scanning, (iii) 3D modeling, (iv) morphometric traits and UPOV characteristics determination by means of 3D image analysis, (v) data analysis by means of discriminant analysis and support vector machine.

The experimental design, the 3D imaging approach, and the overall data analysis scheme is resumed in Figure 2.

## 3 Results

Three‐dimensional image analysis of individual drupes enabled quantification of distinct morphometric traits characterizing the fruit, stone, and internal stone cavity separately. Additionally, we assessed the expression states of the UPOV characteristics (described in the Appendix A) for each drupe. Throughout this work, morphometric traits and UPOV characteristics are prefixed as follows: D for whole fruit, E for stone, and C for internal stone cavity.

### 3.1 Univariate Analysis

The results of univariate statistical analyses are presented separately for morphometric traits (Table 1) and UPOV descriptors (Table 2). No relevant correlations were observed between morphometric traits and the UPOV characteristics not derived from morphometric trait measurements, except for those attributable to inherent geometric relationships (Table S2).

**TABLE 1:** Average values of the morphometric traits (including fruit and stone weights) with standard error in brackets.

Traits	Varieties					No. of sign. diff. variety couples
	Ravece	Ortice	Frantoio	Rotondella	Minucciola	
D_Volume (cm^3^)	3.62 (0.12)	3.3 (0.14)	1.82 (0.1)	2.01 (0.12)	1.78 (0.09)	
	a	a	b	b	b	6
D_Surface area (cm^2^)	12.66 (0.34)	11.82 (0.39)	7.84 (0.29)	8.36 (0.36)	7.58 (0.22)	
	a	a	b	b	b	6
D_Sphericity	0.872 (0.002)	0.870 (0.002)	0.880 (0.002)	0.889 (0.002)	0.891 (0.002)	
	cd	d	bc	ab	a	6
D_L/W*	1.55 (0.01)	1.61 (0.02)	1.47 (0.02)	1.39 (0.03)	1.42 (0.02)	
	ab	a	bc	c	c	5
D_Asymmetry coefficient*	0.58 (0.02)	0.55 (0.04)	0.76 (0.02)	0.65 (0.05)	0.76 (0.03)	
	b	b	a	ab	a	4
D_Position of MTA (% of L)*	−1.32 (0.16)	−1.81 (0.25)	0.29 (0.21)	0.11 (0.26)	−0.35 (0.32)	
	bc	c	a	a	ab	4
D_Weight (g)*	3.47 (0.11)	3.15 (0.15)	1.76 (0.09)	1.9 (0.11)	1.74 (0.11)	
	a	a	b	b	b	6
D_Lenticel density (cm^−2^)*	78.54 (3.57)	76.44 (4.4)	103.01 (4.73)	93.75 (5.13)	118.49 (6.14)	
	c	c	ab	bc	a	5
E_Volume (cm^3^)	0.45 (0.01)	0.46 (0.01)	0.35 (0.01)	0.33 (0.02)	0.23 (0.01)	
	a	a	b	b	c	8
E_Surface area (cm^2^)	3.83 (0.13)	3.9 (0.12)	3.08 (0.12)	2.89 (0.13)	2.38 (0.08)	
	a	a	b	bc	c	7
E_Sphericity	0.743 (0.006)	0.740 (0.007)	0.787 (0.004)	0.796 (0.007)	0.774 (0.012)	
	b	b	a	a	a	6
E_L/W*	2.5 (0.03)	2.61 (0.06)	2.1 (0.03)	1.9 (0.08)	2.14 (0.05)	
	a	a	bc	c	b	7
E_Asymmetry coefficient*	0.39 (0.03)	0.33 (0.03)	0.51 (0.02)	0.46 (0.05)	0.37 (0.03)	
	ab	b	a	ab	ab	1
E_Position of MTA (% of L)*	−0.25 (0.36)	−1.24 (0.35)	−1.22 (0.38)	0.66 (0.63)	−1.01 (1.02)	
	a	a	a	a	a	0
E_Weight (g)*	0.48 (0.02)	0.48 (0.01)	0.38 (0.02)	0.35 (0.02)	0.24 (0)	
	a	a	b	b	c	8
E_Number_of_grooves*	3.75 (0.31)	3.67 (0.37)	2.4 (0.21)	3.88 (0.55)	2.09 (0.28)	
	a	a	ab	a	b	3
C_Volume (mm^3^)	100.02 (4.06)	88.92 (4.01)	72.77 (3.89)	69.67 (6.62)	59.44 (3.94)	
	a	ab	bc	bc	c	6
C_Surface area (mm^2^)	134.33 (3.98)	124.15 (3.92)	105.83 (4.76)	98.5 (5.9)	90.57 (3.78)	
	a	ab	bc	c	c	5
C_Width (mm)	4.92 (0.08)	4.68 (0.08)	4.45 (0.11)	4.76 (0.21)	4.09 (0.11)	
	a	a	ab	a	b	3
C_Sphericity	0.77 (0)	0.77 (0)	0.79 (0)	0.81 (0.01)	0.8 (0)	
	bc	c	abc	a	ab	3
C_Height (mm)	3.84 (0.05)	3.56 (0.08)	3.41 (0.07)	3.54 (0.15)	3.35 (0.85)	
	a	ab	b	ab	b	2
C_L/W	3.18 (0.06)	3.5 (0.09)	3.07 (0.08)	2.61 (0.13)	2.79 (0.09)	
	ab	a	b	c	bc	5
C_Flat	0.78 (0)	0.76 (0.01)	0.77 (0.01)	0.74 (0.01)	0.82 (0.01)	
	ab	b	ab	b	a	2
C_Position of MTA (% of L)	0.64 (0.34)	0.1 (0.32)	0.12 (0.25)	0.69 (0.38)	0 (0.54)	
	a	a	a	a	a	0

*Note:* Values sharing letters on a row are not significantly different (Tukey HSD) at *p* < 0.05. Number of significantly different variety couples in last column reports.

* Traits used to determine states of expression of UPOV characteristics.

**TABLE 2:** Percentages of olives with a given state of expression of UPOV characteristic for each variety.

Characteristic	State of expression	Note	Ravece (%)	Ortice (%)	Frantoio (%)	Rotondella (%)	Minucciola (%)	No. of sign. diff. variety couples
D_Shape*	Ovate	1	3	11	3	6	0	
	Oblong	2	91	81	60	25	36	
	Elliptic	4	6	7	37	63	64	
	Circular	5	0	0	0	0	0	
	Obovate	6	0	0	0	6	0	
	Test		b	b	ab	a	a	4
D_L/W*	Slightly elongated	3	0	0	0	0	0	
	Moderately elongated	5	9	19	40	75	64	
	Very elongated	7	91	81	60	25	36	
	Test		a	a	ab	b	b	4
D_Symmetry*	Symmetric	1	9	15	46	25	27	
	Weakly asymmetric	2	59	44	46	50	73	
	Strongly asymmetric	3	31	41	9	25	0	
	Test		a	a	b	ab	ab	2
D_Weight*	Very low	1	0	0	0	0	0	
	Low	3	0	0	80	75	64	
	Medium	5	88	85	20	25	36	
	High	7	13	15	0	0	0	
	Very high	9	0	0	0	0	0	
	Test		a	a	b	b	b	6
D_Apex	Acute	1	72	67	3	19	18	
	Obtuse	2	28	22	40	31	64	
	Rounded	3	0	11	57	50	18	
	Test		c	c	a	ab	b	7
D_Base	Rounded	1	0	0	9	19	45	
	Rounded to truncate	2	0	0	0	0	0	
	Truncate	3	100	100	91	81	55	
	Test		a	a	a	a	b	4
D_Nipple	Absent or weak	1	63	78	91	100	82	
	Moderate	2	31	22	9	0	18	
	Strong	3	6	0	0	0	0	
	Test		a	ab	ab	b	ab	1
D_Lenticels_number*	Few	1	0	0	0	0	0	
	Medium	2	28	41	14	6	0	
	Many	3	72	59	86	94	100	
	Test		ab	b	ab	ab	a	1
E_Shape*	Ovate	1	3	0	29	13	36	
	Oblong	2	94	85	29	13	36	
	Elliptic	3	3	11	34	44	0	
	Circular	4	0	0	0	0	0	
	Obovate	5	0	4	9	31	27	
	Test		b	b	b	a	ab	3
E_L/W*	Slightly elongated	1	0	0	0	0	0	
	Moderately elongated	2	6	15	71	88	64	
	Very elongated	3	94	85	29	13	36	
	Test		a	a	b	b	b	6
E_Symmetry*	Symmetric	1	0	4	0	6	0	
	Weakly asymmetric	2	28	11	49	31	18	
	Strongly asymmetric	3	72	85	51	63	82	
	Test		a	a	a	a	a	0
E_Weight*	Very low	1	0	0	0	0	0	
	Low	3	6	4	20	31	100	
	Medium	5	19	30	54	56	0	
	High	7	75	67	26	13	0	
	Very high	9	0	0	0	0	0	
	Test		a	a	b	b	c	8
E_Apex	Acute	1	81	89	0	6	27	
	Obtuse	2	19	11	100	94	73	
	Rounded	3	0	0	0	0	0	
	Test		c	c	a	ab	b	7
E_Base	Acute	1	72	89	49	19	45	
	Rounded	2	28	11	49	69	36	
	Truncate	3	0	0	3	13	18	
	Test		bc	b	abc	a	ab	3
E_Rugosity of surface*	Weak	1	56	48	83	50	100	
	Medium	2	34	41	17	38	0	
	Strong	3	9	11	0	13	0	
	Test		a	a	ab	a	b	3
E_mucron	Absent or weak	1	16	a	6	19	9	
	Present	9	84	100	94	81	91	
	Test		a	a	a	a	a	0

*Note:* Olive varieties sharing letters on a row exhibit not significantly (Tukey HSD tests at a *p* < 0.05) different UPOV average notes for a characteristic. The number of significantly different variety couples in the last column reports.

* UPOV characteristics whose states of expression were obtained from morphometric traits measured by 3D image analysis.

#### 3.1.1 Morphometric Traits

In Table 1 are reported the obtained mean values with standard errors for each morphometric trait determined for fruit, stone, and internal stone cavity. Letters indicate statistically significant differences (*p* < 0.05) between varieties. The table also reports the number of variety pairs showing significant differences for each morphometric trait.

E_Volume and E_Weight resulted in the morphometric traits significantly different for 8 olive variety couples, followed by E_Surface area and E_L/W, which were significantly different for 7 ones. The parameters D_Volume, D_Surface area, D_Weight, E_Volume, E_Surface area, E_Sphericity, E_L/W, and E_Weight allowed for distinguishing Ravece and Ortice varieties from the other three varieties, but not each other.

#### 3.1.2 UPOV Characteristics

Table 2 presents, for each variety, the percentages of olives classified into discrete states of expression for each UPOV characteristic, along with letters indicating significant differences in the corresponding average notes among varieties. The number of olive variety pairs exhibiting statistically significant differences for each characteristic is also reported.

The results demonstrate that the UPOV characteristic E_Weight showed significant differences in 8 variety pairs, while D_Apex and E_Apex differed significantly in 7 pairs. Furthermore, the states of expression of D_Weight, D_Apex, E_L/W, E_Weight, and E_Apex in the Ravece and Ortice varieties were significantly different from all other varieties, but not from each other.

The states of expression of D_Shape and D_L/W differentiated Ravece and Ortice only from Rotondella and Minucciola, whereas D_Symmetry distinguished them solely from Frantoio. Additionally, the states of expression of D_Base and E_Weight in Minucciola were significantly different from all other varieties.

### 3.2 Multivariate Analysis

Prior to performing Discriminant Analysis on various data combinations, the Mahalanobis distance provided no presence of multivariate outliers; thus, all olive samples were considered in the analysis.

#### 3.2.1 Preliminary Comparison of Continuous Morphometric Traits Versus Discrete Notes of the UPOV Characteristics as Olive Variety Predictors

The LDA was performed twice to compare results obtained using morphometric traits and UPOV characteristics with strictly corresponding definitions. Such traits and characteristics are indicated by asterisks in Tables 1 and 2. To facilitate comparison, both LDA results are presented in Table 3.

**TABLE 3:** Classification results from LDA using (a) morphometric traits used to determine UPOV notes as predictors and (b) UPOV characteristics derived from morphometric measurements.

	Varieties		Group membership classification					Original
			Ravece	Ortice	Frantoio	Rotondella	Minucciola	
Morphometric traits^a^	Count	Ravece	25	4	2	1	0	32
		Ortice	6	18	2	1	0	27
		Frantoio	1	0	31	1	2	35
		Rotondella	0	2	3	11	0	16
		Minucciola	0	0	2	0	9	11
	%	Ravece	**78.1**	12.5	6.3	3.1	0.0	100.0
		Ortice	22.2	**66.7**	7.4	3.7	0.0	100.0
		Frantoio	2.9	0.0	**88.6**	2.9	5.7	100.0
		Rotondella	0.0	12.5	18.8	**68.8**	0.0	100.0
		Minucciola	0.0	0.0	18.2	0.0	**81.8**	100.0
UPOV characteristics^b^	Count	Ravece	25	6	0	0	1	32
		Ortice	14	12	1	0	0	27
		Frantoio	3	0	30	0	2	35
		Rotondella	1	0	5	8	2	16
		Minucciola	0	0	1	2	8	11
	%	Ravece	**78.1**	18.8	0.0	0.0	3.1	100.0
		Ortice	51.9	**44.4**	3.7	0.0	0.0	100.0
		Frantoio	8.6	0.0	**85.7**	0.0	5.7	100.0
		Rotondella	6.3	0.0	31.3	**50.0**	12.5	100.0
		Minucciola	0.0	0.0	9.1	18.2	**72.7**	100.0

*Note:* Bold values indicate percent of correctly classified olives of each variety.

^a^ 77.7% of olive fruits correctly classified, overall, by using morphometric traits.

^b^ 68.6% of olive fruits correctly classified, overall, by using UPOV characteristics.

The LDA based on continuous morphometric traits achieved an overall correct classification of 77.7% of olive fruits. The highest classification accuracy was observed for Frantoio (88.6%), followed by Minucciola (81.8%), Ravece (78.1%), Rotondella (68.8%), and Ortice (66.7%). Consistent with univariate analysis, LDA revealed greater similarity between Ortice and Ravece, with 12.5% of Ortice olives misclassified as Ravece and 22.2% of Ravece olives misclassified as Ortice. Additionally, 18.8% of Rotondella olives were misclassified as Frantoio. Cross‐validation using the leave‐one‐out approach (Table S3) yielded an overall 68.6% correct prediction.

When the notes corresponding to the discrete states of expression of the UPOV characteristics were used as predictors, the overall correct classification was 68.6%. The highest classification accuracy was again observed for Frantoio (85.7%), followed by Ravece (78.1%), Minucciola (72.7%), Rotondella (50.0%), and Ortice (44.4%). The results confirmed the difficulty in distinguishing Ravece and Ortice, with 51.9% of Ortice olives misclassified as Ravece and 18.8% of Ravece olives misclassified as Ortice. Furthermore, 31.3% of Rotondella olives were misclassified as Frantoio. Cross‐validation (Table S4) resulted in a 47.9% overall correct prediction.

#### 3.2.2 Olive Variety Classification Combining Morphometric Traits and Visual UPOV Characteristics

Discriminant Analysis (DA) performed using all morphometric traits combined with the notes of the visual UPOV characteristics (i.e., those not marked with an asterisk in Table 2) achieved an overall correct classification of 88.4% (Table 4). Specifically, 100% of Frantoio olives were correctly classified, followed by Minucciola (90.9%), Ravece (84.4%), Ortice (81.5%), and Rotondella (81.3%) ones.

**TABLE 4:** Classification results from LDA using fruit, stone, and cavity morphometric traits and visual UPOV characteristics (notes) as predictors.

Varieties		Group membership classification					Original
		Ravece	Ortice	Frantoio	Rotondella	Minucciola	
Count	Ravece	27	4	1	0	0	32
	Ortice	3	22	2	0	0	27
	Frantoio	0	0	35	0	0	35
	Rotondella	0	1	2	13	0	16
	Minucciola	0	0	1	0	10	11
%	Ravece	**84.4**	12.5	3.1	0.0	0.0	100.0
	Ortice	11.1	**81.5**	7.4	0.0	0.0	100.0
	Frantoio	0.0	0.0	**100.0**	0.0	0.0	100.0
	Rotondella	0.0	6.3	12.5	**81.3**	0.0	100.0
	Minucciola	0.0	0.0	9.1	0.0	**90.9**	100.0

*Note:* 88.4% of olive fruits correctly classified, overall. Bold values indicate percent of correctly classified olives of each variety.

Also using such a combination of predictors, Ortice and Ravece remained the most similar varieties, with 12.5% of Ortice olives misclassified as Ravece and 11.1% of Ravece olives misclassified as Ortice. Additionally, 12.5% of Rotondella olives were misclassified as Frantoio. Cross‐validation using the leave‐one‐out method (Table S5) yielded a 70.2% overall correct prediction.

To evaluate the relative contribution of different olive parts to varietal classification, we conducted additional DAs using distinct combinations of the morphometric traits and visual UPOV characteristics: those of fruit and stone (excluding stone cavity), and those of fruit, stone, and cavity analyzed separately.

Using combined fruit and stone data (excluding stone cavity data), the overall correct classification decreased from 88.4% to 83.5%. Analysis based on fruit, stone, and cavity data used separately yielded overall classifications of 72.7%, 71.9%, and 59.5%, respectively. Complete discriminant scores and leave‐one‐out cross‐validation results for all LDA are provided in Tables S6–S13.

#### 3.2.3 Olive Variety Overlapping

The Bhattacharyya coefficient (*BhC*) was computed for each variety pair as a varietal overlapping indicator, using the discriminant function four‐dimensional datasets derived from LDA performed with all morphometric traits combined with visual UPOV characteristics. This calculation was repeated using discriminant function values obtained from DAs based on all possible combinations of data from different olive parts (fruit [D], stone [E], and cavity [C]). Results are presented in Table 5.

**TABLE 5:** Bhattacharyya coefficients of discriminant scores in DF^4^ domain for each couple of olive varieties and all data combinations.

Variety couple	Data Combinations*				
	D + E + C	D + E	D	E	C
Ravece‐Ortice	0.427	0.520	0.857	0.599	0.803
Ravece‐Frantoio	0.045	0.068	0.048	0.164	0.548
Ravece‐Rotondella	0.064	0.089	0.097	0.270	0.227
Ravece‐Minucciola	0.035	0.071	0.534	0.268	0.199
Ortice‐Frantoio	0.046	0.089	0.186	0.071	0.444
Ortice‐Rotondella	0.067	0.098	0.138	0.194	0.196
Ortice‐Minucciola	0.037	0.147	0.416	0.144	0.133
Frantoio‐Rotondella	0.212	0.300	0.615	0.417	0.377
Frantoio‐Minucciola	0.111	0.149	0.392	0.236	0.407
Minucciola‐Rotondella	0.203	0.257	0.480	0.345	0.247
Average	0.125	0.179	0.376	0.271	0.358

* D = fruit, E = stone, C = stone cavity.

The lowest average *BhC* value was achieved when using data from all olive structures (D + E + C), followed by the combined data of fruit and stone (D + E). Among analyses of individual structures, stone data (E) yielded the lowest average *BhC*; although the minimum *BhC* for specific variety pairs could occur with either fruit (D), stone (E), or cavity (C) data depending on the pair.

Employing all available data (D + E + C), the Ravece‐Minucciola pair exhibited the lowest *BhC*, while Ravece‐Ortice showed the highest. This latter result for Ravece‐Ortice was consistent across all data combinations, whereas Ravece‐Minucciola's lowest *BhC* was only confirmed using combined fruit and stone data (D + E).

*BhC* values exceeding 0.5 were observed for Ravece‐Frantoio (with stone cavity data), for Ravece‐Minucciola and Frantoio‐Rotondella (with fruit data).

#### 3.2.4 Olive Variety Predictors Rank

To assess the relative importance of the 29 predictors (morphometric traits and visual UPOV characteristics) for varietal differentiation and classification in the DA, we calculated potency indices from the LDA results. The resulting predictor ranking is presented in Table 6.

**TABLE 6:** Predictors rank in olive variety discriminant analysis based on potency index.

Rank	Predictors	Potency Index	Rank	Predictors	Potency Index
1	E_Apex	0.233	16	D_lenticel_density	0.049
2	D_Volume	0.223	17	E_Base	0.037
3	D_Weight	0.214	18	D_Base	0.034
4	D_Surface area	0.207	19	D_Asymmetry coefficient	0.034
5	E_L/W	0.144	20	E_Asymmetry coefficient	0.024
6	D_Apex	0.105	21	C_Width	0.023
7	E_Surface area	0.079	22	C_Height	0.023
8	D_Position of MTA	0.078	23	E_Number_of_grooves	0.023
9	E_Sphericity	0.075	24	C_Sphericity	0.022
10	D_Sphericity	0.074	25	D_nipple	0.018
11	E_Volume	0.070	26	E_Position of MTA	0.012
12	D_L/W	0.058	27	C_Flat	0.012
13	C_Surface	0.057	28	E_Mucron	0.008
14	C_Volume	0.050	29	C_Position of MTA	0.004
15	C_L/W	0.050			

E_Apex emerged as the most discriminant predictor for distinguishing among the five olive varieties, followed by D_Volume and D_Weight. In contrast, E_Mucron and C_Position of MTA ranked as the least discriminant predictors. Notably, all stone cavity related predictors ranked below position 10 in importance.

#### 3.2.5 Support Vector Machine Learning—Olive Variety Prediction Combining Morphometric Traits and Visual UPOV Characteristics

The predictive potential of the combination of all morphometric traits and visual UPOV characteristics of fruits and stones for olive variety classification was evaluated by using an SVM model. The training results are reported in the Table S14.

The SVM model achieved peak performance with a linear kernel function, demonstrating 94.05% classification accuracy during training (Table S14). When applied to the validation dataset, it correctly predicted varieties for 67.57% of olive samples (Table 7). Ortice variety showed the highest prediction accuracy (87.5%), followed by Ravece (70%), Minucciola (66.7%) and Rotondella (60%) varieties. Frantoio variety showed the lowest prediction accuracy (54.5%).

**TABLE 7:** Variety prediction results with SVM. 30% of olives without variety tags as validation dataset. All morphometric traits and visual UPOV characteristics were considered as predictors.

Varieties		Predicted group membership					Original
		Ravece	Ortice	Frantoio	Rotondella	Minucciola	
Count	Ravece	7	2	0	0	1	10
	Ortice	1	7	0	0	0	8
	Frantoio	2	0	6	3	0	11
	Rotondella	0	0	2	3	0	5
	Minucciola	1	0	0	0	2	3
%	Ravece	70.0	20.0	0.0	0.0	10.0	100.0
	Ortice	12.5	87.5	0.0	0.0	0.0	100.0
	Frantoio	18.2	0.0	54.5	27.3	0.0	100.0
	Rotondella	0.0	0.0	40.0	60.0	0.0	100.0
	Minucciola	33.3	0.0	0.0	0.0	66.7	100.0

*Note:* 67.57% of olive fruits correctly predicted, overall.

## 4 Discussion

The X‐ray micro‐CT scanning methodology employed in this study enabled the accurate reconstruction of both the external morphology of individual olive fruits and the external and internal structures of olive stones across the five examined varieties. 3D imaging of the mesocarp, endocarp (stone), and endocarp cavity was obtained through a single scan per fruit, eliminating the need for time‐consuming pulp removal and preserving structural integrity for comprehensive 3D analysis.

Morphometric traits for each olive fruit part were quantified using image analysis, with reference to the morphological characteristics outlined in the UPOV guidelines for *Olea europaea* L. (UPOV 2011). Additionally, states of expression of these UPOV characteristics were computationally derived. Differently from the conventional UPOV protocol, which assesses states of expression of the UPOV characteristics based on bulk fruit groups, the application of image analysis to individual fruits facilitated the precise determination of the morphometric traits and of the corresponding notes. This approach minimized human operator bias and enabled a robust statistical evaluation of all recorded parameters.

Univariate analysis (Tables 1 and 2) revealed that no single morphometric trait or UPOV characteristic could fully discriminate all studied olive varieties. However, between 1 and 8 variety pairs exhibited statistically significant differences depending on the specific trait or UPOV characteristic, with the exceptions of E_Position of MTA, C_Position of MTA, E_Symmetry, and E_mucron, which showed no significant variation across varieties. Notably, the morphometric traits D_Volume, D_Surface area, D_Weight, E_Volume, E_Surface area, E_Sphericity, E_L/W, and E_Weight, along with the visual UPOV characteristics D_Apex and E_Apex, distinguished the Ravece and Ortice varieties from the other three, but not each other. On the other hand, these two varieties exhibited significantly larger fruit dimensions and more elongated stone morphologies with respect to the other three varieties.

Multivariate analysis based on DA, comparing morphometric traits and the states of expression of corresponding UPOV characteristics (Table 3), demonstrated better olive variety classification using morphometric traits rather than UPOV characteristics. The decline in classification accuracy using UPOV characteristics was particularly evident for the Ortice and Rotondella varieties. On the other hand, univariate analysis (Tables 1 and 3) consistently showed that the number of significantly different variety pairs was never higher for UPOV characteristics compared to the corresponding morphometric traits. These results directly stem from the discretization process, in which notes are assigned to the states of expression of UPOV characteristics instead of utilizing continuous measured data obtained using image analysis. Consequently, we propose that the UPOV guidelines (UPOV 2022) concerning image analysis (e.g., TGP/8/5: Part II:11: “Examining Characteristics Using Image Analysis”) should be reconsidered. Specifically, an explicit recommendation could be introduced to discourage the use of notes when analyzing characteristics through image analysis. Exceptions could be made only for characteristics that do not demand high precision and are more challenging to determine automatically than by visual observation. In this study, characteristics related to the apex and base of both fruit and stone have been identified as belonging to this latter category, where visual assessment may be preferable. On the other hand, such characteristics have been found not correlated with morphometric traits (Table S2), thus such visually estimated parameters add new, not redundant, information useful for the variety classification.

A high classification accuracy (approaching 90%) was achieved using a combination of morphometric traits and visual UPOV characteristics related to olive apex and base morphology (Table 4). Correct varietal classification ranged from 81.3% for Rotondella to 100% for Frantoio. Notably, no misclassifications occurred for Rotondella or Minucciola, whereas at least one olive from each variety was erroneously classified as Frantoio.

The Bhattacharyya coefficient (BC) (Ray 1989) was employed to quantify overlap between all pairwise combinations of olive varieties, as it is an effective metric for comparing multivariate distributions in statistics, machine learning, and data science (e.g., Van Molle et al. 2021; Patra et al. 2015), including applications in discriminant analysis (e.g., Guo et al. 2022). Here, the BC was also used to assess the contribution of different olive fruit parts to varietal overlap (Table 7).

The highest degree of overlap was observed between Ravece and Ortice when considering all morphological data, quantitatively confirming their phenotypic similarity, as previously suggested by univariate analyses. When evaluating individual fruit parts, endocarp (stone) data yielded the lowest average varietal overlap. This aligns with prior studies (e.g., Martínez et al. 2018; Manolikaki et al. 2022; Blazakis et al. 2024), which posit that endocarp morphology is less influenced by environmental and agronomic factors compared to mesocarp traits, making it a more reliable indicator for cultivar discrimination. The endocarp's relative stability likely stems from its lignified structure, protective pulp encasement, and limited environmental exposure during development (D'Imperio et al. 2011).

Notably, BC values derived from datasets of all olive parts (including stone cavity data) consistently exhibited lower varietal overlap—both on average and for each pairwise comparison—than those excluding stone cavity information. This underscores the diagnostic value of internal stone cavity morphology in varietal differentiation.

Conceptually, the BC inversely relates to the “distinctness” criterion outlined in UPOV guidelines for new variety testing (UPOV 2022). However, unlike UPOV protocols, the BC provides an objective measure of dissimilarity, eliminating subjectivity in weights assignment to the characteristics required by UPOV (2022). We therefore propose its integration into UPOV frameworks to enhance the reproducibility and rigor of “distinctness” assessments.

A predictor ranking was derived by calculating the potency index from the multivariate LDA results (Table 6). This index effectively integrates two key aspects of discriminative capacity: (1) the number of olive varieties significantly differentiated by each predictor, and (2) the average statistical significance of these differences. The potency index values exhibit a trend similar to that of the product of these two metrics (Tables 3 and 4; Supporting Information S1), confirming its robustness as a composite measure of varietal discrimination. Thus, the ranking provides a reliable assessment of each morphological parameter's overall discriminatory capacity, reflecting both the extent (“how many” varieties are distinguished) and degree (“how much” separation occurs) of differentiation.

Notably, E_Apex emerged as the top‐ranked predictor, aligning with recent studies employing distinct methodologies. Miho et al. (2023) and Blazakis et al. (2024)—using deep learning architectures and meta‐classifiers, respectively—similarly identified endocarp extremities as critical regions for varietal classification. This convergence of evidence is further supported by traditional morphological taxonomy, where expert evaluations prioritize endocarp apex and base traits (Barranco et al. 2000; UPOV 2011). The consistency across computational and human‐driven approaches underscores the biological and diagnostic significance of these morphological features.

To evaluate the predictive potential of olive fruit and stone morphology for varietal classification, a SVM model was trained on 70% of the available data and validated on the remaining 30%. The optimal model performance was achieved using a linear kernel function. The SVM demonstrated higher classification accuracy (94.05%) compared to LDA (88.41%), with only marginally lower correct varietal prediction accuracy (67.57% vs. 70.22%)—despite being trained on a reduced dataset and validated on an independent subset. These results indicate that SVM is a robust tool for olive varietal prediction, even with relatively small sample sizes, as in this study. In contrast, LDA proved more suitable for elucidating the underlying morphological discriminants of classification (i.e., the “why” behind the results).

The multivariate analysis of olive morphological traits presented here holds significant promise for antifraud applications. This approach could be implemented across supply chain checkpoints—from initial post‐harvest stages to olive oil milling—to verify the authenticity of monocultivar oils and safeguard their quality standards.

## 5 Conclusions

X‐ray micro‐CT on olive fruits enabled accurate three‐dimensional morphological characterization of whole fruit architecture, including endocarp structure and internal stone cavity morphology, without requiring physical removal of fruit pulp and stone sectioning.

SVM model based on morphological traits from all fruit parts achieved high varietal prediction accuracy, even with limited sample sizes. LDA provided comprehensive evaluation of the contribution of individual morphological traits to variety classification and a detailed comparison among the different cultivars. Rotondella and Minucciola emerged as the most morphologically distinct varieties, whereas Frantoio showed the highest degree of overlap, being all other varieties misclassified as Frantoio at least once. Ortice and Ravece exhibited the greatest similarity, frequently confounding one another while remaining distinct from other cultivars. This similarity was primarily attributable to their larger fruit and stone dimensions.

Bhattacharyya coefficient analysis confirmed the predominant role of endocarp traits in varietal discrimination. Notably, while endocarp cavity parameters did not rank among the top predictors, their inclusion consistently enhanced discriminant performance, suggesting their complementary value in cultivar identification. Moreover, the Bhattacharyya coefficient, when applied to discriminant functions, emerged as an efficient and easily interpretable parameter for quantifying pairwise varietal distinctness. This finding can provide a valuable contribution to the UPOV standard procedures, for example, integrating the Bhattacharyya coefficient into UPOV “distinctness” protocols as an objective, quantitative index, capable of enhancing the rigor and reproducibility of variety certification protocols. Moreover, continuous morphometric data derived from image analysis demonstrated superior discriminative power compared to categorical UPOV characteristics, underscoring the need for methodological updates in varietal characterization standards. The adoption of such morphometric data in the standard certification procedures could modernize germplasm characterization, aligning it with precision agriculture and digital phenotyping advancements.

The demonstrated ability to differentiate cultivars solely through fruit and stone morphology presents significant potential for anti‐fraud checks in the post‐harvest stage, before olive milling. Thus, the proposed approach could effectively be applied to safeguard the authenticity of high‐quality monocultivar oils, particularly for protected designations of origin varieties.

Overall considered, olive fruit phenotyping using 3D image analysis could accelerate breeding efforts by enabling high‐resolution trait analysis, supporting the development of improved olive cultivars. Regulatory bodies and certification agencies could adopt the 3D morphometric traits for more objective and reproducible varietal assessment, reducing reliance on expert panels and improving the authenticity verification in the olive oil supply chain.

## Author Contributions

**Laura Gargiulo:** formal analysis (equal), investigation (equal), visualization (lead), writing – original draft (equal), writing – review and editing (supporting). **Antonio Gaetano Napolitano:** formal analysis (equal), investigation (equal), visualization (equal). **Sabrina Maria Marsala:** formal analysis (equal), investigation (equal), visualization (supporting). **Anna Di Salle:** data curation (supporting), resources (lead). **Giacomo Mele:** conceptualization (lead), funding acquisition (lead), investigation (equal), methodology (lead), supervision (lead), writing – original draft (equal), writing – review and editing (lead).

## Conflicts of Interest

The authors declare no conflicts of interest.

## Supporting information


**Data S1:** fsn370797‐sup‐0001‐Supplementary Figure S1.jpg.

**Data S2:** fsn370797‐sup‐0002‐Supplementary tables.docx.

## Figures and Tables

**FIGURE A1:**
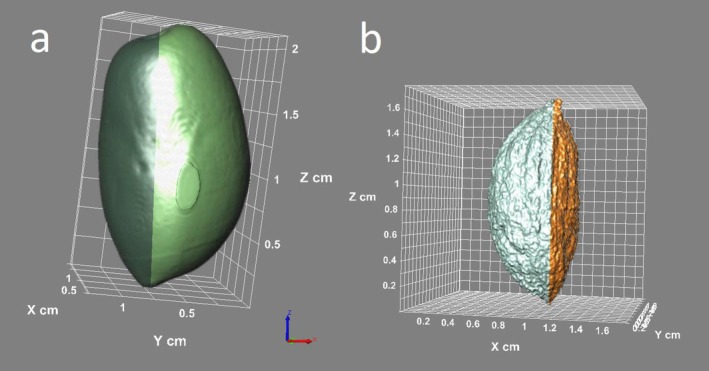
Volumes for asymmetry coefficient measurement. Couples of volumes of a fruit (a) and a stone (b) identified for the determination of the asymmetry coefficient.

**FIGURE A2:**
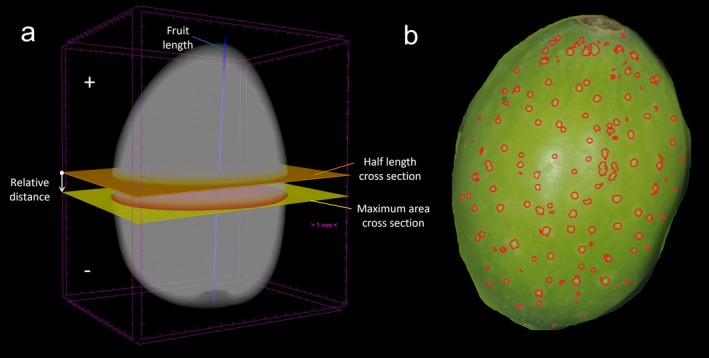
Position of maximum transverse area and lenticel density measurement. (a) Geometrical elements for the determination of the position of maximum transverse area. Distances toward the apex are positive. An example of a fruit with a negative relative distance. (b) Automatic object counting for the determination of the lenticel density.

**FIGURE A3:**
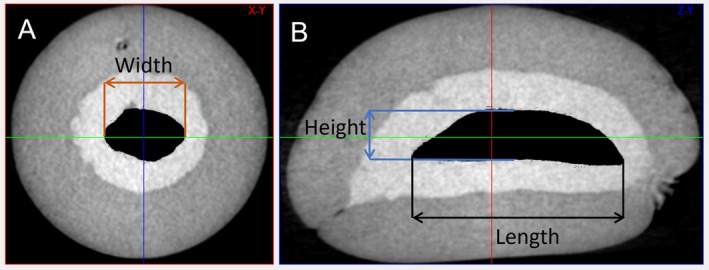
Stone cavity traits. Some reference measurements were carried out on the stone cavity. (A) Largest transversal section. (B) Largest longitudinal section.

## Data Availability

Original raw image dataset of all 121 olive fruits scanned and analyzed in this study is freely available at Mendeley Data repository (DOI: 10.17632/49y4zjx9tj.2).

## Appendix A

X‐ray micro‐CT imaging of olive drupes and three‐dimensional image analysis enabled quantification of the morphometric parameters listed in Table A1. These parameters were systematically categorized into three distinct groups based on their anatomical reference: whole fruit traits (prefix: D), endocarp (stone) traits (prefix: E), and endocarp cavity traits (prefix: C).

The Asymmetry coefficient was calculated as the minimum volume ratio between the two partitions created by any plane within a sheaf of planes intersecting the base‐apex axis of the fruit, stone, or stone cavity. This coefficient represents the most asymmetric division possible along the longitudinal axis of the structure. Figure A1 provides a visual demonstration of these partitioned volumes for both a whole fruit and a stone.

The Position of Maximum Transverse Area (MTA) was quantified as the longitudinal distance between the transverse cross‐section with maximum area and the mid‐length cross‐section of the analyzed structure (fruit, stone, or stone cavity). The Position of Maximum Transverse Area (MTA) shows a positive value if the MTA is toward the fruit apex (see Figure A2a). This trait provides a measure of longitudinal asymmetry in olive morphology.

Lenticel density was calculated using the following protocol:

1. Automated counting of lenticels from pre‐scanning photographic images (Figure A2b).
2. Surface area measurement of each fruit hemisphere via 3D image analysis.
3. Density calculation as: lenticel count/(total fruit surface area/2).

The 3D imaging allowed to measure morphometric traits of the cavity of the stones, as shown in Figure A3.

The E_weight was determined from stone volume without cavity by means of preliminary measurements of stone tissue density.

In addition, the characteristics suggested by the guidelines for testing distinctness, uniformity, and stability of olive varieties of the UPOV (2011) have been determined. They are reported in Table A2. Except for D_Apex, D_Base, D_Nipple, E_Apex, E_Base, and E_Mucron, which were qualitatively evaluated on the 3D reconstructed model of fruits, all remaining characteristics were measured by using three‐dimensional image analysis, different from the UPOV original definition. This required applying threshold values on measured traits to assign the different states of expression defined by UPOV to a given characteristic. Each state of expression is allocated a corresponding numerical “note” (UPOV 2020) whose differences are conventionally used to determine phenotypic distance between each pair of varieties in UPOV distinctness tests (UPOV 2022).

**TABLE A1:** List of the morphometric traits* determined for whole fruit, stone, and cavity of stone of each olive.

Type	Morphometric trait (unit)	Definition
Fruit	D_Volume (cm^3^)	Volume of whole fruit
	D_Surface (cm^2^)	Outer surface of fruit
	D_Sphericity (−)	$\sqrt[{3}]{\pi {\left(6\cdot D\_\text{Volume}\right)}^{2}}/D\_\text{Surface}$
	D_L/W (−)	Fruit length/Fruit width
	D_Asymmetry coefficient (−)	Minimum ratio between the couples of volumes into which a sheaf of planes passing through the line joining the base and apex divides a fruit.
	D_Position of MTA (% of fruit length)	Distance between the fruit cross‐sections corresponding to Maximum Transverse Area (MTA) and half length. Positive value if the MTA is toward fruit apex.
	D_Weight (g)	Weight of the whole fruit
	D_Lenticel density (cm^−2^)	Number of lenticel/fruit surface
Stone	E_Volume (cm^3^)	Volume of whole stone
	E_Surface (cm^2^)	Outer surface of stone
	E_Sphericity (−)	$\sqrt[{3}]{\pi {\left(6\cdot E\_\text{Volume}\right)}^{2}}/E\_\text{Surface}$
	E_L/W (−)	Length of stone/Width of stone
	E_Asymmetry coefficient (−)	Minimum ratio between the couples of volumes into which a sheaf of planes passing through the line joining the base and apex divides a stone
	E_Position of MTA (% of stone length)	Distance between the stone cross‐sections corresponding to MTA and half length. Positive value if the MTA is toward stone apex
	E_Weight (g)	Weight of the stone
	E_Number of grooves (−)	Number of grooves that can be seen all around the stone surface
Stone cavity	C_Volume (mm^3^)	Volume of the whole internal cavity of the stone
	C_Surface (mm^2^)	Outer surface of the cavity
	C_Width (mm)	Width of stone cavity
	C_Sphericity (−)	$\sqrt[{3}]{\pi {\left(6\cdot C\_\text{Volume}\right)}^{2}}/C\_\text{Surface}$
	C_Height (mm)	Height of the stone cavity
	C_L/W (−)	C_lenght/C_width
	C_Flat (−)	C_height/C_width
	C_Position of MTA (% of cavity length)	Distance between the cavity cross‐sections corresponding to MTA and half length. Positive value if the MTA is toward cavity apex

* Fruit and endocarp weights are also included in the above list.

**TABLE A2:** Characteristics for olive fruits and stones with their properties as originally defined by UPOV.

Characteristics	States of expression	Numerical notes	Type of observation/record*	Type of expression**
D_shape	Ovate Oblong Elliptic Circular Obovate	1 2 4 5 6	V/G	PQ
D_L/W	Slightly elongated Moderately elongated Very elongated	3 5 7	V/G	QN
D_Symmetry	Symmetric Weakly asymmetric Strongly asymmetric	1 2 3	V/G	QN
D_Weight	Very low Low Medium High Very high	1 3 5 7 9	M/G	QN
D_Apex	Acute Obtuse Rounded	1 2 3	V/G	PQ
D_Base	Rounded Rounded to truncate Truncate	1 2 3	V/G	PQ
D_Nipple	Absent or weak Moderate Strong	1 2 3	V/G	QL
D_Lenticels number	Few Medium Many	1 2 3	V/G	QN
E_Shape	Ovate Oblong Elliptic Circular Obovate	1 2 3 4 5	V/G	PQ
E_L/W	Slightly elongated Moderately elongated Very elongated	1 2 3	V/G	QN
E_Symmetry	Symmetric Weakly asymmetric Strongly asymmetric	1 2 3	V/G	QN
E_Weight	Very low Low Medium High Very high	1 3 5 7 9	M/G	QN
E_Apex	Acute Obtuse Rounded	1 2 3	V/G	PQ
E_Base	Acute Rounded Truncate	1 2 3	V/G	PQ
E_Rugosity of surface	Weak Medium Strong	1 2 3	V/G	QN
E_Mucron	Absent Present	1 9	V/G	QL

* V = visual, M = measurement, /G = for a group of drupes, /S = for single drupes.

** QN = quantitative, QL = qualitative, PQ = pseudo qualitative.
