# Supplementary figures and images for: Olive Variety Classification and Prediction From 3D Morphology of Fruit and Stone: A Study Case on Five South Italy Autochthone Cultivars
Source: Food Sci Nutr. 2025 Aug 31;13(9):e70797. doi: 10.1002/fsn3.70797 (PMC12399264; doi:10.1002/fsn3.70797)

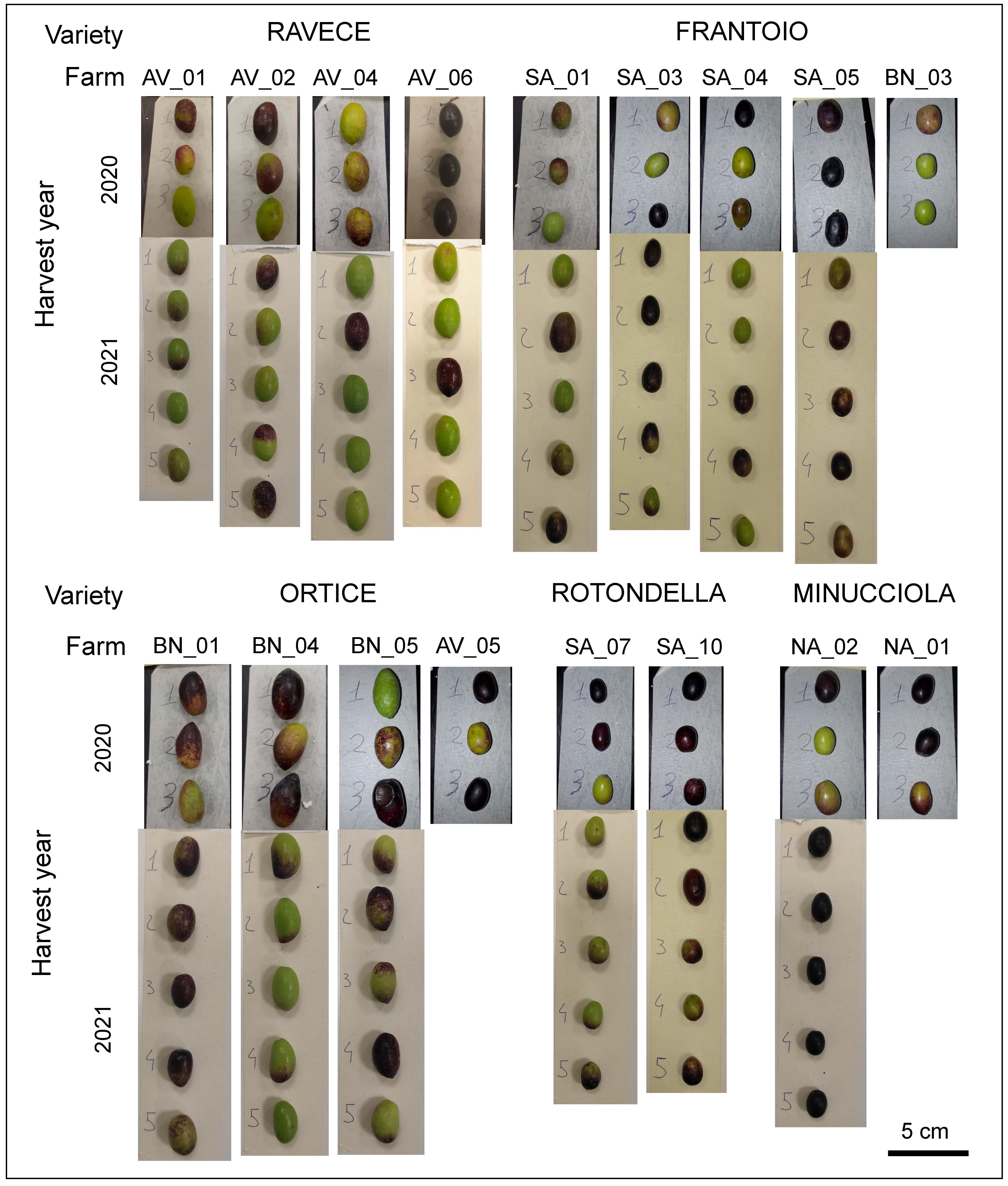

Supplement: Supplementary file 1 — Data S1: fsn370797‐sup‐0001‐Supplementary Figure S1.jpg. [file FSN3-13-e70797-s001.jpg]
