# Supplementary material for: Olive Variety Classification and Prediction From 3D Morphology of Fruit and Stone: A Study Case on Five South Italy Autochthone Cultivars
Source: Food Sci Nutr. 2025 Aug 31;13(9):e70797. doi: 10.1002/fsn3.70797 (PMC12399264; doi:10.1002/fsn3.70797)
Supplement: Supplementary file 2 — Data S2: fsn370797‐sup‐0002‐Supplementary tables.docx. [file FSN3-13-e70797-s002.docx]

**Supplementary Tables**

**Supplementary Table S1.** Geographical coordinates (WGS84) of the farms involved in the study.

| Olive variety | Farm code | Geographical area | Latitude | Longitude |
| --- | --- | --- | --- | --- |
| Ravece | AV_01 | Areale_Arianese | 41.169718 | 15.123061 |
|  | AV_02 | Areale_della_Baronia | 41.063888 | 15.148893 |
|  | AV_04 | Valle_Alto_Calore | 40.920725 | 15.093192 |
|  | AV_06 | Valle_Calore | 41.01986 | 15.025001 |
| Ortice | AV_05 | Valle_Caudina | 41.047078 | 14.68831 |
|  | BN_01 | Titerno | 41.300502 | 14.686115 |
|  | BN_04 | Colline_del_medio_calore | 41.09308 | 14.841754 |
|  | BN_05 | Alto_Tammaro | 41.376936 | 14.790829 |
| Frantoio | BN_03 | Taburno | 41.087326 | 14.533495 |
|  | SA_01 | Tanagro | 40.584279 | 15.406084 |
|  | SA_03 | Tanagro_zona_bassa | 40.614499 | 15.386465 |
|  | SA_04 | Tanagro | 40.496079 | 15.529225 |
|  | SA_05 | Alto_e_medio_sele | 40.715356 | 15.251142 |
| Rotondella | SA_07 | Calore_Salernitano | 40.454028 | 15.194335 |
|  | SA_10 | Monti_Picentini | 40.716942 | 14.931672 |
| Minucciola | NA_01 | Penisola_Sorrentina | 40.668942 | 14.440554 |
|  | NA_02 | Penisola_Sorrentina | 40.613328 | 14.381533 |

**Supplementary Table S2.** Pearson correlation coefficients (p-values below) among the morphometric traits and the notes of the UPOV characteristics. In yellow the values higher than 0,9 and in orange the values lower than 0,9 but higher than 0,8.

**Supplementary Table S3.** “Leave one out” cross-validation results from DA using morphometric traits corresponding to UPOV characteristics as predictors.

| Varieties | | Predicted Group Membership | | | | | Original |
| --- | --- | --- | --- | --- | --- | --- | --- |
|  |  | Ravece | Ortice | Frantoio | Rotondella | Minucciola |  |
| Count | Ravece | 23 | 6 | 2 | 1 | 0 | 32 |
|  | Ortice | 10 | 13 | 2 | 2 | 0 | 27 |
|  | Frantoio | 2 | 0 | 29 | 2 | 2 | 35 |
|  | Rotondella | 0 | 2 | 4 | 10 | 0 | 16 |
|  | Minucciola | 0 | 0 | 3 | 0 | 8 | 11 |
| % | Ravece | 71,9 | 18,8 | 6,3 | 3,1 | ,0 | 100,0 |
|  | Ortice | 37,0 | 48,1 | 7,4 | 7,4 | ,0 | 100,0 |
|  | Frantoio | 5,7 | ,0 | 82,9 | 5,7 | 5,7 | 100,0 |
|  | Rotondella | ,0 | 12,5 | 25,0 | 62,5 | ,0 | 100,0 |
|  | Minucciola | ,0 | ,0 | 27,3 | ,0 | 72,7 | 100,0 |
| 68,6% of olive fruits correctly predicted, overall. Cross validation is done only for those varieties in the analysis. In cross validation, each variety is predicted by the functions derived from all varieties other than that variety. | | | | | | | |

**Supplementary Table S4.** “Leave one out” cross -validation results from DA using UPOV characteristics (notes) determined from morphometric traits as predictors.

| Varieties | | Predicted Group Membership | | | | | Original |
| --- | --- | --- | --- | --- | --- | --- | --- |
|  |  | Ravece | Ortice | Frantoio | Rotondella | Minucciola |  |
| Count | Ravece | 18 | 11 | 0 | 1 | 2 | 32 |
|  | Ortice | 21 | 4 | 1 | 1 | 0 | 27 |
|  | Frantoio | 4 | 0 | 25 | 3 | 3 | 35 |
|  | Rotondella | 2 | 0 | 4 | 7 | 3 | 16 |
|  | Minucciola | 0 | 0 | 4 | 3 | 4 | 11 |
| % | Ravece | 56,3 | 34,4 | ,0 | 3,1 | 6,3 | 100,0 |
|  | Ortice | 77,8 | 14,8 | 3,7 | 3,7 | ,0 | 100,0 |
|  | Frantoio | 11,4 | ,0 | 71,4 | 8,6 | 8,6 | 100,0 |
|  | Rotondella | 12,5 | ,0 | 25,0 | 43,8 | 18,8 | 100,0 |
|  | Minucciola | ,0 | ,0 | 36,4 | 27,3 | 36,4 | 100,0 |
| 47,9% of olive fruits correctly predicted, overall Cross validation is done only for those varieties in the analysis. In cross validation, each variety is predicted by the functions derived from all varieties other than that variety. | | | | | | | |

**Supplementary Table S5.** “Leave one out” cross-validation results from DA using all morphometric traits and visual UPOV characteristics (notes) as predictors.

| Varieties | | Predicted Group Membership | | | | | Original |
| --- | --- | --- | --- | --- | --- | --- | --- |
|  |  | Ravece | Ortice | Frantoio | Rotondella | Minucciola |  |
| Count | Ravece | 19 | 8 | 2 | 1 | 2 | 32 |
|  | Ortice | 5 | 19 | 3 | 0 | 0 | 27 |
|  | Frantoio | 2 | 1 | 29 | 2 | 1 | 35 |
|  | Rotondella | 0 | 1 | 2 | 11 | 2 | 16 |
|  | Minucciola | 1 | 1 | 1 | 1 | 7 | 11 |
| % | Ravece | 59,4 | 25,0 | 6,3 | 3,1 | 6,3 | 100,0 |
|  | Ortice | 18,5 | 70,4 | 11,1 | ,0 | ,0 | 100,0 |
|  | Frantoio | 5,7 | 2,9 | 82,9 | 5,7 | 2,9 | 100,0 |
|  | Rotondella | ,0 | 6,3 | 12,5 | 68,8 | 12,5 | 100,0 |
|  | Minucciola | 9,1 | 9,1 | 9,1 | 9,1 | 63,6 | 100,0 |
| 70,2% of olive fruits correctly predicted, overall. Cross validation is done only for those varieties in the analysis. In cross validation, each variety is predicted by the functions derived from all varieties other than that variety. | | | | | | | |

**Supplementary Table S6.** Classification results from DA using all morphometric traits and visual UPOV characteristics of fruits and stones (without stone cavity traits) as predictors.

| Varieties | | Group Membership Classification | | | | | Original |
| --- | --- | --- | --- | --- | --- | --- | --- |
|  |  | Ravece | Ortice | Frantoio | Rotondella | Minucciola |  |
| Count | Ravece | 25 | 5 | 1 | 1 | 0 | 32 |
|  | Ortice | 2 | 22 | 3 | 0 | 0 | 27 |
|  | Frantoio | 1 | 0 | 32 | 2 | 0 | 35 |
|  | Rotondella | 0 | 0 | 2 | 13 | 1 | 16 |
|  | Minucciola | 0 | 1 | 1 | 0 | 9 | 11 |
| % | Ravece | 78,1 | 15,6 | 3,1 | 3,1 | ,0 | 100,0 |
|  | Ortice | 7,4 | 81,5 | 11,1 | ,0 | ,0 | 100,0 |
|  | Frantoio | 2,9 | ,0 | 91,4 | 5,7 | ,0 | 100,0 |
|  | Rotondella | ,0 | ,0 | 12,5 | 81,3 | 6,3 | 100,0 |
|  | Minucciola | ,0 | 9,1 | 9,1 | ,0 | 81,8 | 100,0 |
| 83,5% of olive fruits correctly classified, overall. | | | | | | | |

**Supplementary Table S7.** “Leave one out” cross-validation results from DA using all morphometric traits and visual UPOV characteristics of fruits and stones (without stone cavity traits) as predictors.

| Varieties | | Predicted Group Membership | | | | | Original |
| --- | --- | --- | --- | --- | --- | --- | --- |
|  |  | Ravece | Ortice | Frantoio | Rotondella | Minucciola |  |
| Count | Ravece | 19 | 7 | 3 | 3 | 0 | 32 |
|  | Ortice | 6 | 18 | 3 | 0 | 0 | 27 |
|  | Frantoio | 1 | 0 | 29 | 5 | 0 | 35 |
|  | Rotondella | 0 | 2 | 2 | 10 | 2 | 16 |
|  | Minucciola | 1 | 1 | 2 | 2 | 5 | 11 |
| % | Ravece | 59,4 | 21,9 | 9,4 | 9,4 | ,0 | 100,0 |
|  | Ortice | 22,2 | 66,7 | 11,1 | ,0 | ,0 | 100,0 |
|  | Frantoio | 2,9 | ,0 | 82,9 | 14,3 | ,0 | 100,0 |
|  | Rotondella | ,0 | 12,5 | 12,5 | 62,5 | 12,5 | 100,0 |
|  | Minucciola | 9,1 | 9,1 | 18,2 | 18,2 | 45,5 | 100,0 |
| 66,9% of olive fruits correctly predicted, overall. Cross validation is done only for those varieties in the analysis. In cross validation, each variety is predicted by the functions derived from all varieties other than that variety. | | | | | | | |

**Supplementary Table S8.** Classification results from DA using all morphometric traits and qualitative UPOV characteristics of only fruits (without stone and stone cavity traits) as predictors.

| Varieties | | Group Membership classification | | | | | Original |
| --- | --- | --- | --- | --- | --- | --- | --- |
|  |  | Ravece | Ortice | Frantoio | Rotondella | Minucciola |  |
| Count | Ravece | 23 | 6 | 2 | 1 | 0 | 32 |
|  | Ortice | 4 | 20 | 3 | 0 | 0 | 27 |
|  | Frantoio | 2 | 0 | 31 | 1 | 1 | 35 |
|  | Rotondella | 0 | 0 | 8 | 7 | 1 | 16 |
|  | Minucciola | 0 | 0 | 4 | 0 | 7 | 11 |
| % | Ravece | 71,9 | 18,8 | 6,3 | 3,1 | ,0 | 100,0 |
|  | Ortice | 14,8 | 74,1 | 11,1 | ,0 | ,0 | 100,0 |
|  | Frantoio | 5,7 | ,0 | 88,6 | 2,9 | 2,9 | 100,0 |
|  | Rotondella | ,0 | ,0 | 50,0 | 43,8 | 6,3 | 100,0 |
|  | Minucciola | ,0 | ,0 | 36,4 | ,0 | 63,6 | 100,0 |
| 72,7% of olive fruits correctly classified, overall. | | | | | | | |

**Supplementary Table S9.** “Leave one out” cross-validation results from DA using all morphometric traits and qualitative UPOV characteristics of only fruits (without stone and stone cavity traits) as predictors.

| Varieties | | Predicted Group Membership | | | | | Original |
| --- | --- | --- | --- | --- | --- | --- | --- |
|  |  | Ravece | Ortice | Frantoio | Rotondella | Minucciola |  |
| Count | Ravece | 22 | 7 | 2 | 1 | 0 | 32 |
|  | Ortice | 9 | 15 | 3 | 0 | 0 | 27 |
|  | Frantoio | 2 | 1 | 25 | 4 | 3 | 35 |
|  | Rotondella | 0 | 0 | 8 | 5 | 3 | 16 |
|  | Minucciola | 0 | 0 | 5 | 2 | 4 | 11 |
| % | Ravece | 68,8 | 21,9 | 6,3 | 3,1 | ,0 | 100,0 |
|  | Ortice | 33,3 | 55,6 | 11,1 | ,0 | ,0 | 100,0 |
|  | Frantoio | 5,7 | 2,9 | 71,4 | 11,4 | 8,6 | 100,0 |
|  | Rotondella | ,0 | ,0 | 50,0 | 31,3 | 18,8 | 100,0 |
|  | Minucciola | ,0 | ,0 | 45,5 | 18,2 | 36,4 | 100,0 |
| 58,7% of olive fruits correctly predicted, overall. Cross validation is done only for those varieties in the analysis. In cross validation, each variety is predicted by the functions derived from all varieties other than that variety. | | | | | | | |

**Supplementary Table S10.** Classification results from DA using all morphometric traits and qualitative UPOV characteristics of only stones (without cavity traits) as predictors.

| Varieties | | Group Membership classification | | | | | Original |
| --- | --- | --- | --- | --- | --- | --- | --- |
|  |  | Ravece | Ortice | Frantoio | Rotondella | Minucciola |  |
| Count | Ravece | 20 | 6 | 4 | 1 | 1 | 32 |
|  | Ortice | 6 | 18 | 2 | 1 | 0 | 27 |
|  | Frantoio | 0 | 0 | 30 | 2 | 3 | 35 |
|  | Rotondella | 0 | 1 | 3 | 12 | 0 | 16 |
|  | Minucciola | 2 | 0 | 2 | 0 | 7 | 11 |
| % | Ravece | 62,5 | 18,8 | 12,5 | 3,1 | 3,1 | 100,0 |
|  | Ortice | 22,2 | 66,7 | 7,4 | 3,7 | ,0 | 100,0 |
|  | Frantoio | ,0 | ,0 | 85,7 | 5,7 | 8,6 | 100,0 |
|  | Rotondella | ,0 | 6,3 | 18,8 | 75,0 | ,0 | 100,0 |
|  | Minucciola | 18,2 | ,0 | 18,2 | ,0 | 63,6 | 100,0 |
| 71,9% of olive fruits correctly classified, overall. | | | | | | | |

**Supplementary Table S11.** “Leave one out” cross -validation results from DA using all morphometric traits and qualitative UPOV characteristics of only stones (without cavity traits) as predictors.

| Varieties | | Predicted Group Membership | | | | | Original |
| --- | --- | --- | --- | --- | --- | --- | --- |
|  |  | Ravece | Ortice | Frantoio | Rotondella | Minucciola |  |
| Count | Ravece | 14 | 11 | 4 | 2 | 1 | 32 |
|  | Ortice | 9 | 15 | 2 | 1 | 0 | 27 |
|  | Frantoio | 0 | 0 | 28 | 4 | 3 | 35 |
|  | Rotondella | 0 | 1 | 6 | 9 | 0 | 16 |
|  | Minucciola | 3 | 0 | 3 | 2 | 3 | 11 |
| % | Ravece | 43,8 | 34,4 | 12,5 | 6,3 | 3,1 | 100,0 |
|  | Ortice | 33,3 | 55,6 | 7,4 | 3,7 | ,0 | 100,0 |
|  | Frantoio | ,0 | ,0 | 80,0 | 11,4 | 8,6 | 100,0 |
|  | Rotondella | ,0 | 6,3 | 37,5 | 56,3 | ,0 | 100,0 |
|  | Minucciola | 27,3 | ,0 | 27,3 | 18,2 | 27,3 | 100,0 |
| 57% of olive fruits correctly predicted, overall. Cross validation is done only for those varieties in the analysis. In cross validation, each variety is predicted by the functions derived from all varieties other than that variety. | | | | | | | |

**Supplementary Table S12.** Classification results from DA using all morphometric traits of only cavities as predictors.

| Varieties | | Group Membership classification | | | | | Original |
| --- | --- | --- | --- | --- | --- | --- | --- |
|  |  | Ravece | Ortice | Frantoio | Rotondella | Minucciola |  |
| Count | Ravece | 21 | 6 | 3 | 0 | 2 | 32 |
|  | Ortice | 8 | 17 | 1 | 1 | 0 | 27 |
|  | Frantoio | 5 | 3 | 22 | 2 | 3 | 35 |
|  | Rotondella | 0 | 1 | 4 | 11 | 0 | 16 |
|  | Minucciola | 2 | 0 | 7 | 1 | 1 | 11 |
| % | Ravece | 65,6 | 18,8 | 9,4 | ,0 | 6,3 | 100,0 |
|  | Ortice | 29,6 | 63,0 | 3,7 | 3,7 | ,0 | 100,0 |
|  | Frantoio | 14,3 | 8,6 | 62,9 | 5,7 | 8,6 | 100,0 |
|  | Rotondella | ,0 | 6,3 | 25,0 | 68,8 | ,0 | 100,0 |
|  | Minucciola | 18,2 | ,0 | 63,6 | 9,1 | 9,1 | 100,0 |
| 59,5% of olive fruits correctly classified, overall | | | | | | | |

**Supplementary Table S13.** “Leave one out” cross -validation results from DA using all morphometric traits of only cavities as predictors.

| Varieties | | Predicted Group Membership | | | | | Original |
| --- | --- | --- | --- | --- | --- | --- | --- |
|  |  | Ravece | Ortice | Frantoio | Rotondella | Minucciola |  |
| Count | Ravece | 21 | 6 | 3 | 0 | 2 | 32 |
|  | Ortice | 8 | 17 | 1 | 1 | 0 | 27 |
|  | Frantoio | 5 | 3 | 22 | 2 | 3 | 35 |
|  | Rotondella | 0 | 1 | 4 | 11 | 0 | 16 |
|  | Minucciola | 2 | 0 | 7 | 1 | 1 | 11 |
| % | Ravece | 65,6 | 18,8 | 9,4 | ,0 | 6,3 | 100,0 |
|  | Ortice | 29,6 | 63,0 | 3,7 | 3,7 | ,0 | 100,0 |
|  | Frantoio | 14,3 | 8,6 | 62,9 | 5,7 | 8,6 | 100,0 |
|  | Rotondella | ,0 | 6,3 | 25,0 | 68,8 | ,0 | 100,0 |
|  | Minucciola | 18,2 | ,0 | 63,6 | 9,1 | 9,1 | 100,0 |
| 54,5% of olive fruits correctly predicted, overall. Cross validation is done only for those varieties in the analysis. In cross validation, each variety is predicted by the functions derived from all varieties other than that variety. | | | | | | | |

**Table S14.** Results of SVM training using 70% of all olives with variety tagged. All morphometric traits and visual UPOV characteristics were considered as predictors.

| Varieties | | Group Membership classification | | | | | Original |
| --- | --- | --- | --- | --- | --- | --- | --- |
|  |  | Ravece | Ortice | Frantoio | Rotondella | Minucciola |  |
| Count | Ravece | 20 | 1 | 1 | 0 | 0 | 22 |
|  | Ortice | 2 | 16 | 1 | 0 | 0 | 19 |
|  | Frantoio | 0 | 0 | 24 | 0 | 0 | 24 |
|  | Rotondella | 0 | 0 | 0 | 11 | 0 | 11 |
|  | Minucciola | 0 | 0 | 0 | 0 | 8 | 8 |
| % | Ravece | 90,9 | 4,5 | 4,5 | ,0 | ,0 | 100,0 |
|  | Ortice | 10,5 | 84,2 | 5,3 | ,0 | ,0 | 100,0 |
|  | Frantoio | ,0 | ,0 | 100,0 | ,0 | ,0 | 100,0 |
|  | Rotondella | ,0 | ,0 | ,0 | 100,0 | ,0 | 100,0 |
|  | Minucciola | ,0 | ,0 | ,0 | ,0 | 100,0 | 100,0 |
| Accuracy of training: 94,05% | | | | | | | |
